# Preliminary observations of glucose metabolism dysregulation in pediatric Huntington’s disease

**DOI:** 10.3389/fneur.2025.1626275

**Published:** 2025-08-20

**Authors:** Federica Graziola, Federica Rachele Danti, Martina Penzo, Antonio Spagarino, Eleonora Minacapilli, Marco Moscatelli, Federica Zibordi, Caterina Mariotti, Giovanna Zorzi

**Affiliations:** ^1^Department of Pediatric Neuroscience, Fondazione IRCCS Istituto Neurologico Carlo Besta, Milan, Italy; ^2^Department of Biomedical and Clinical Sciences, Postgraduate School of Child Neuropsychiatry, University of Milan, Milan, Italy; ^3^Pediatric Section, Department of Medical Sciences, University of Ferrara, Ferrara, Italy; ^4^Neuroradiology Unit, Fondazione IRCCS Istituto Neurologico Carlo Besta, Milan, Italy; ^5^Medical Genetics and Neurogenetics Unit, Fondazione IRCCS Istituto Neurologico Carlo Besta, Milan, Italy

**Keywords:** Huntington’s disease, pediatric Huntington’s disease, juvenile-onset HD, GLUT-1 deficiency syndrome, GLUT1

## Abstract

**Background:**

Pediatric Huntington’s disease (PHD), a rare and severe form of juvenile-onset Huntington’s disease (JOHD), is associated with highly expanded CAG repeats in the *HTT* gene and a rapidly progressive neurodegenerative course. Recent studies have suggested that glucose metabolism may be impaired in PHD due to reduced expression of glucose transporters in the brain, resembling aspects of GLUT1 Deficiency Syndrome (GLUT1DS).

**Methods:**

We investigated glucose metabolism in two pediatric patients with genetically confirmed PHD (CAG repeats: 76 and 79) referred to our tertiary care center. Clinical, neuroimaging, and neuropsychological data were collected alongside metabolic assessments, including cerebrospinal fluid (CSF) and plasma glucose and lactate levels, CSF-to-serum glucose ratio, and red blood cell GLUT1 expression using the METAglut1 test. 18F-FDG PET imaging and brain MRI were performed to assess cerebral metabolism and structural changes.

**Results:**

Both patients exhibited progressive motor and cognitive decline with dystonia-parkinsonian features, learning disabilities, and behavioral disturbances. Brain MRI showed caudate and putaminal atrophy, while PET imaging demonstrated severely reduced glucose uptake in the basal ganglia. CSF/plasma glucose ratios were within or near the lower end of the normal range (0.51 and 0.6), and GLUT1 expression in red blood cells was within normal limits. No significant biochemical alterations consistent with GLUT1DS were detected.

**Conclusion:**

Our findings confirm localized cerebral hypometabolism in the basal ganglia of PHD patients, consistent with previous neuropathological reports. However, systemic biochemical indicators of glucose transport deficiency, including erythrocyte GLUT1 function and CSF glucose, were not significantly altered. While glucose dysregulation appears to be a feature of PHD brain pathology, our results do not support the use of metabolic interventions such as the ketogenic diet in the absence of confirmed GLUT1 dysfunction. Further studies in larger cohorts are warranted to better characterize the metabolic profile of PHD and guide therapeutic strategies.

## 1 Introduction

Huntington’s disease (HD) is an autosomal dominant neurodegenerative disorder caused by an unstable expansion of CAG repeats in the *HTT* gene, leading to an elongated polyglutamine tract in the Huntingtin protein. Larger CAG expansions correlate with earlier onset of symptoms. Typically, adult-onset HD presents around the age of 40, and is characterized by neurological, behavioral, and cognitive decline, along with involuntary movements such as chorea (1).

When HD manifests before the age of 21 years, it is classified as juvenile-onset HD (JOHD), with the subset of cases occurring in younger children referred to as pediatric Huntington disease (PHD). JOHD represents 5–10% of all HD cases, but the exact prevalence of PHD remains uncertain (2). Severe forms of PHD, particularly in children with over 70–80 CAG repeats, are associated with rapid progression, reduced lifespan, motor and developmental delays, severe dystonia (without chorea), learning difficulties, seizures, and atypical brain abnormalities (2, 3). Behavioral issues such as hyperactivity and aggression further complicate the clinical spectrum of JOHD and PHD (4).

Recent research by Tramutola et al. (5) have identified a dysfunctional hypometabolic state in PHD brains with highly expanded CAG repeats. By analyzing the expression of Glucose Transporter 1 (GLUT1) and Glucose Transporter 3 (GLUT3) proteins in PHD, JOHD, and adult-onset HD patients compared to controls, they observed a significant reduction in GLUT1 and GLUT3 levels in the frontal cortex and fibroblasts of PHD patients with highly expanded mutations. GLUT1 and GLUT3 are uniporter proteins encoded by the *SLC2A1* and *SLC2A3* (solute carrier family 2, facilitated glucose transporter member 1 and 3) genes, respectively. These transporters facilitate glucose transport across the plasma membranes of mammalian cells. The findings align with evidence of mitochondrial dysfunction in HD (6), supporting the hypothesis of impaired energy metabolism in these patients.

In particular, deficits in electron transport chain activity, reduced oxidative phosphorylation capacity, and impaired tricarboxylic acid (TCA) cycle function have been observed (7, 8). These findings align with functional neuroimaging studies in HD patients showing early and progressive cerebral hypometabolism, even in pre-symptomatic stages, accompanied by increased lactate levels and ATP depletion in both brain and peripheral tissues (9). Mechanistically, mutant huntingtin impairs mitochondrial function through downregulation of PGC-1α, a transcriptional coactivator critical for controlling mitochondrial biogenesis, respiration, and other metabolic pathways (10).

Interestingly, the metabolic abnormalities observed in PHD mirror those seen in GLUT1 Deficiency Syndrome (GLUT1DS), a rare neurological disorder caused by mutations in the SLC2A1 gene. GLUT1DS is characterized by seizures, developmental delays, motor impairments, and movement disorders. Its treatment relies on the ketogenic diet (KD), a high-fat, low-carbohydrate diet that induces ketosis, providing an alternative energy source to glucose (5, 11).

## 2 Methods

We present two patients referring to the Department of Pediatric Neuroscience of the Istituto Neurologico Carlo Besta, Milan with highly expanded CAG repeats in the *HTT* gene and a diagnosis of PHD. Molecular analysis was performed on DNA sample extracted from peripheral blood by using the Amplidex PCR/CE HTT kit (Asuragen®). The number of triplets was determined by electrophoresis on an ABI-PRISM 3500 XL DX capillary sequencer.

Clinical data were gathered through detailed medical and family histories, comprehensive neurological examinations, and neuropsychological testing. The clinical assessments included the Unified Huntington’s Disease Rating Scale Total Motor Scale (UHDRS-TMS). The Instrumental assessment comprised brain MRI and a positron emission tomography (PET) using 18\u00B0F-fluorodeoxyglucose (18 FDG).

The metabolic investigations included cerebrospinal fluid (CSF) glucose and lactate and plasma glucose and lactate. We conducted a simultaneous lumbar puncture and venous blood sampling, performed according to standard clinical protocols. In addition, the serum to CSF glucose ratio was calculated. Erythrocyte glucose uptake was assessed by GLUT1 Quantification on Red Blood Cells assay (The METAglut1 test METAFORA Biosystems®) (12).

Informed consent was obtained from the patients’ guardians for the processing of personal data and publication.

## 3 Results

### 3.1 Clinical features

Patient 1: a 13-year-old girl diagnosed with pediatric Huntington’s disease (CAG 79 repeats) at age 12, with family history positive for the disease (father, grandfather, and great-aunt affected). Pregnancy and delivery were uneventful, psychomotor and language development were normal though she has had right foot internal rotation since age 6, treated with physiotherapy. Learning difficulties were noted from age 6, along with a progressive decline in motor and cognitive functions. Neurological findings include dystonia-parkinsonian syndrome and dysarthria. Although she follows a regular diet, she experiences swallowing difficulties, particularly with liquids. Cognitive assessment reveals borderline intellectual functioning with deficits in visual-motor integration, attention, verbal fluency, working memory, and processing speed. She also experiences social anxiety and struggles with peer relationships. At age 12, she began treatment with L-Dopa (300 mg/day) with minor improvement in motor fluidity and started trihexyphenidyl (6 mg/day) at age 13. She attends weekly speech therapy. Currently she is in her third year of middle school. A home educator and psychological support are planned.

Patient 2: A 16-year-old boy diagnosed with pediatric Huntington’s disease (CAG 76 repeats) at age 15. He is an only child, with an affected biological father and a healthy mother. His pregnancy was complicated by placental detachment, toxoplasmosis, and emergency C-section, although there was no perinatal distress. Psychomotor and language development were normal. From the age of 8, he began to experience academic difficulties, and at the age of 10 was diagnosed with mixed learning and motor disabilities (dysgraphia). His motor skills gradually deteriorated, with worsening coordination, frequent falls, executive dysfunction, and abnormal eye movements. Neurologically, he presented with ophthalmoplegia, dysarthric speech, bradykinesia, and dystonia. Moreover, he developed progressive swallowing difficulties, particularly with liquids and solids, leading to reduced caloric intake and respiratory issues. He is on a semi-solids and semi-liquids diet. Cognitive evaluation at age 15 showed a mild intellectual disability, with marked difficulties in working memory and processing speed but relatively preserved verbal comprehension and visuospatial reasoning. He Also exhibited attention and visuomotor integration difficulties, aggressivity (especially toward his mother), and poor frustration tolerance. He started L-Dopa (200 mg/day) at age 14, with initial improvement, and trihexyphenidyl (12 mg/day) was added at age 15. Currently he is in his second year of high school and practicing speech and physiotherapy.

**Figure 1:**
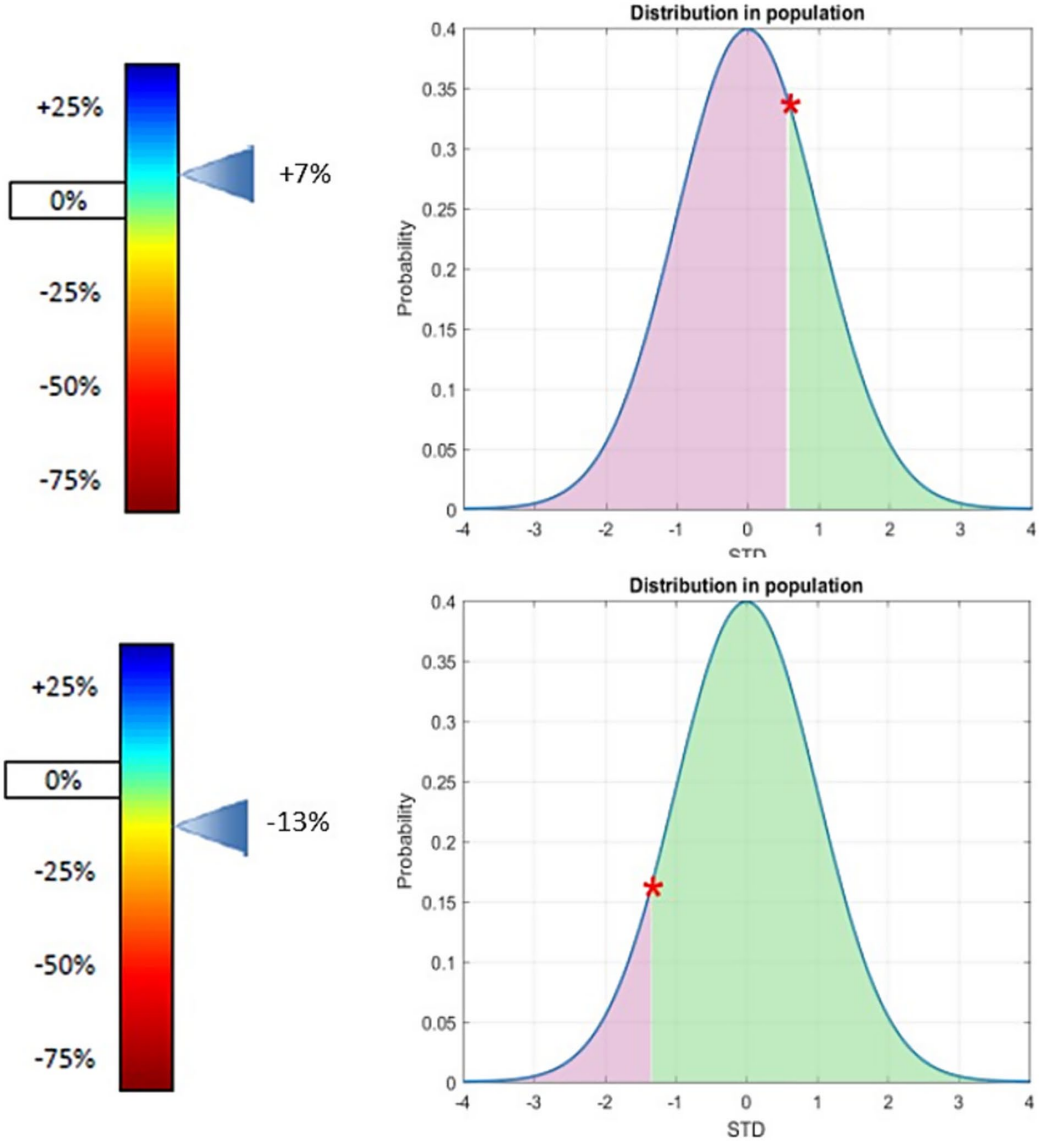
GLUT1 expression at the patient’s red blood cell membrane (relative to population mean) in patient one (top) and two (bottom). Variation of GLUT1 expression at the patients’ red blood cell membrane (relative to population mean) position of the patient in the general population. Both patients show that there is no significant difference of the expression of GLUT1 on red blood cells according to our test compared to the general population. Patients confirmed Glut1DS have a deficit of Glut1 expression on the surface of their red blood cells, most often between −60% and −21% the cut-off of positivity is −24%. For further detail of the METAglut1 test see reference (12).

**Figure 2:**
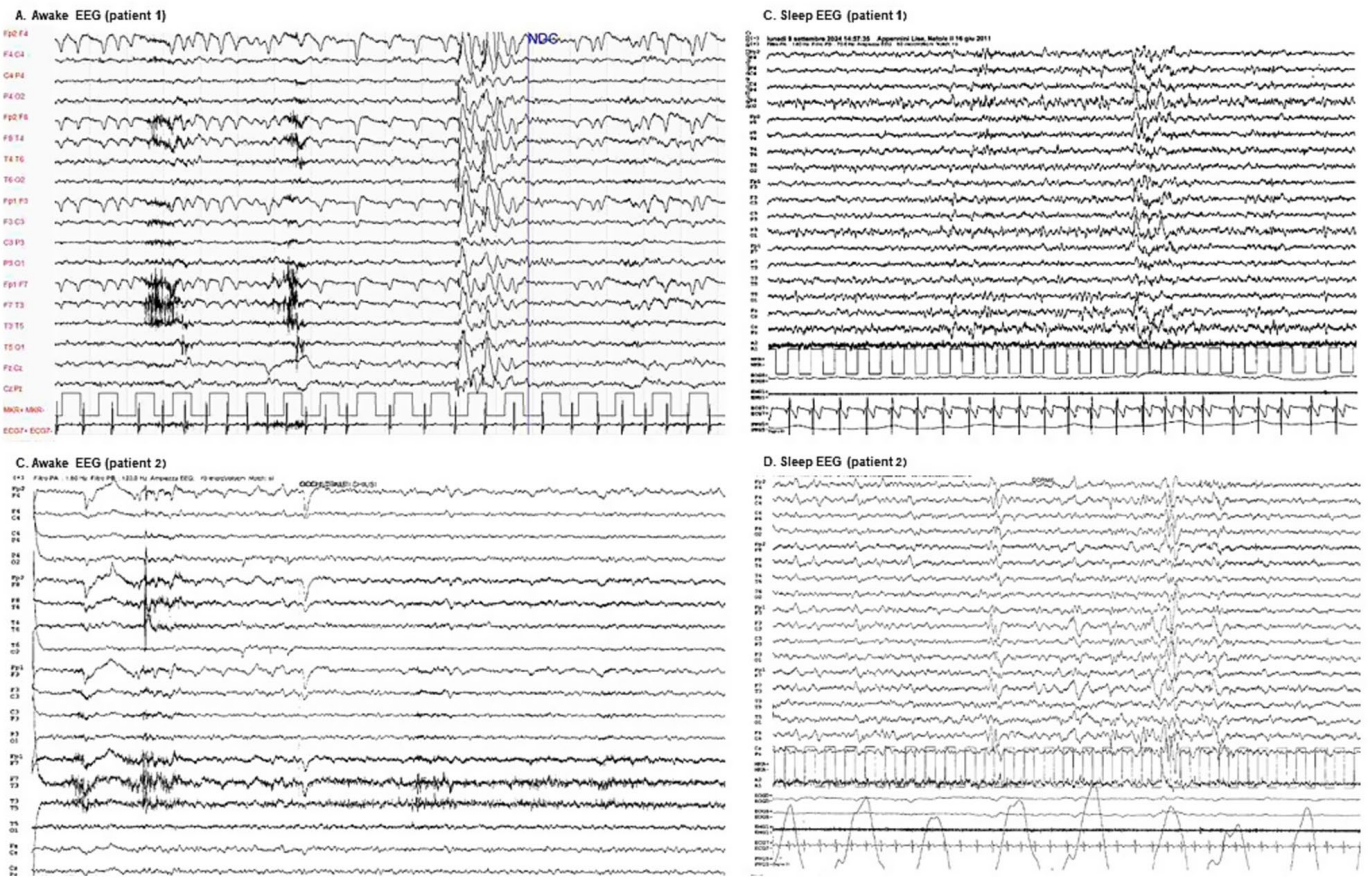
Awake and sleep EEGs. **(A)** Patient 1 awake EEG: demonstrates bilateral posterior theta slowing (5–6 Hz, low to moderate amplitude) alongside anterior low-voltage beta activity, accompanied by ocular artifacts. Following hyperventilation, a single brief generalized high-amplitude slow spike-and-wave discharge, predominantly anterior, is observed without any clinical correlate. **(B)** Patient 1 sleep EEG: exhibits a well-organized sleep pattern with symmetrical physiological waveforms. Brief episodes of diffuse sharp-wave bursts predominantly localized to anterior regions are present, appearing without associated clinical correlates. **(C)** Patient 2 awake EEG: reveals a globally low-voltage and disorganized background activity, characterized by diffuse theta–beta frequencies with limited reactivity. Eye closure induces irregular posterior low-alpha rhythms intermixed with faster frequencies. **(D)** Patient 2 sleep EEG: displays a recognizable but irregular NREM sleep pattern, marked by bilateral and symmetric hypnic graphoelements with a monomorphic and simplified morphology.

For further clinical details and investigation, see Table 1 and Figures 1, 2.

**Table 1:** Clinical and demographic data.

	Patient 1	Patient 2
Gender	Female	Male
Age last evaluation (y)	13	16
CAG repeats	79	76
Inheritance	Paternal	Paternal
Age of onset (y)	6	8
Age at diagnosis (y)	12	15
Symptoms at onset	Learning disabilities and right foot dystonia	Learning disabilities
Current clinical symptoms	Dystonia-parkinsonism, dysarthria and speech disorders, cognitive decline	Dystonia-parkinsonism, dysarthria and speech disorders, cognitive decline, oculomotor apraxia, dysphagia
HD phase	Moderate	Moderate
UHDRS-TMS	42/124	47/124
Brain MRI	Atrophy in caudate nuclei and putamen with signal abnormalities	Hyperintensities and reduced volume in the putamen and caudate nuclei, with dorsolateral profile changes
EEG	Bilateral posterior theta slowing alongside anterior low-voltage beta activity, accompanied by ocular artifacts. Following hyperventilation, a single brief generalized high-amplitude slow spike-and-wave discharge, predominantly anterior, is observed without any clinical correlate.	Globally low-voltage and disorganized background activity, characterized by diffuse theta–beta frequencies with limited reactivity. Eye closure induces irregular posterior low-alpha rhythms intermixed with faster frequencies
Sleep EEG	Well-organized sleep pattern with symmetrical physiological waveforms. Brief episodes of diffuse sharp-wave bursts predominantly localized to anterior regions are present, appearing without associated clinical correlates	Irregular NREM sleep pattern, marked by bilateral and symmetric hypnic graphoelements with a monomorphic and simplified morphology.
Medical therapy	L-dopa (300 mg/die) Trihexyphenidyl (6 mg/die)	L-dopa (200 mg / die) Trihexyphenidyl (12 mg /die)
Blood glucose (mg/dL) Ref. range: 50–110	115	97
CSF glucose (mg/dL) Ref. range:40–80	59	62
Blood/CSF glucose ratio Ref. range in GLUT1DS < 0.4 with a range of 0.19–0.59	0.51	0.60
CSF Lactate (μmoli/l) Ref. range: 800–2,100	1,127	1,528
METAglu1 test Variation of GLUT1 expression at the patient’s red blood cell membrane relative to population mean	+7% (+ 5 sd)	−13% (−1.3 sd)

y, years; HD, Huntington’s disease; UHDRS-TMS, unified Huntington’s disease rating scale total motor score, MRI, magnetic resonance imaging; CSF, cerebrospinal fluid; sd, standard deviation.

### 3.2 Metabolic assessment

To verify the hypothesis of a dysregulation of glucose metabolism, we assessed CSF and plasma glucose level with the CSF/plasma ratios resulting within or near the lower normal range, erythrocyte GLUT1 expression within population reference intervals, and normal lactate CSF levels.

**Figure 3:**
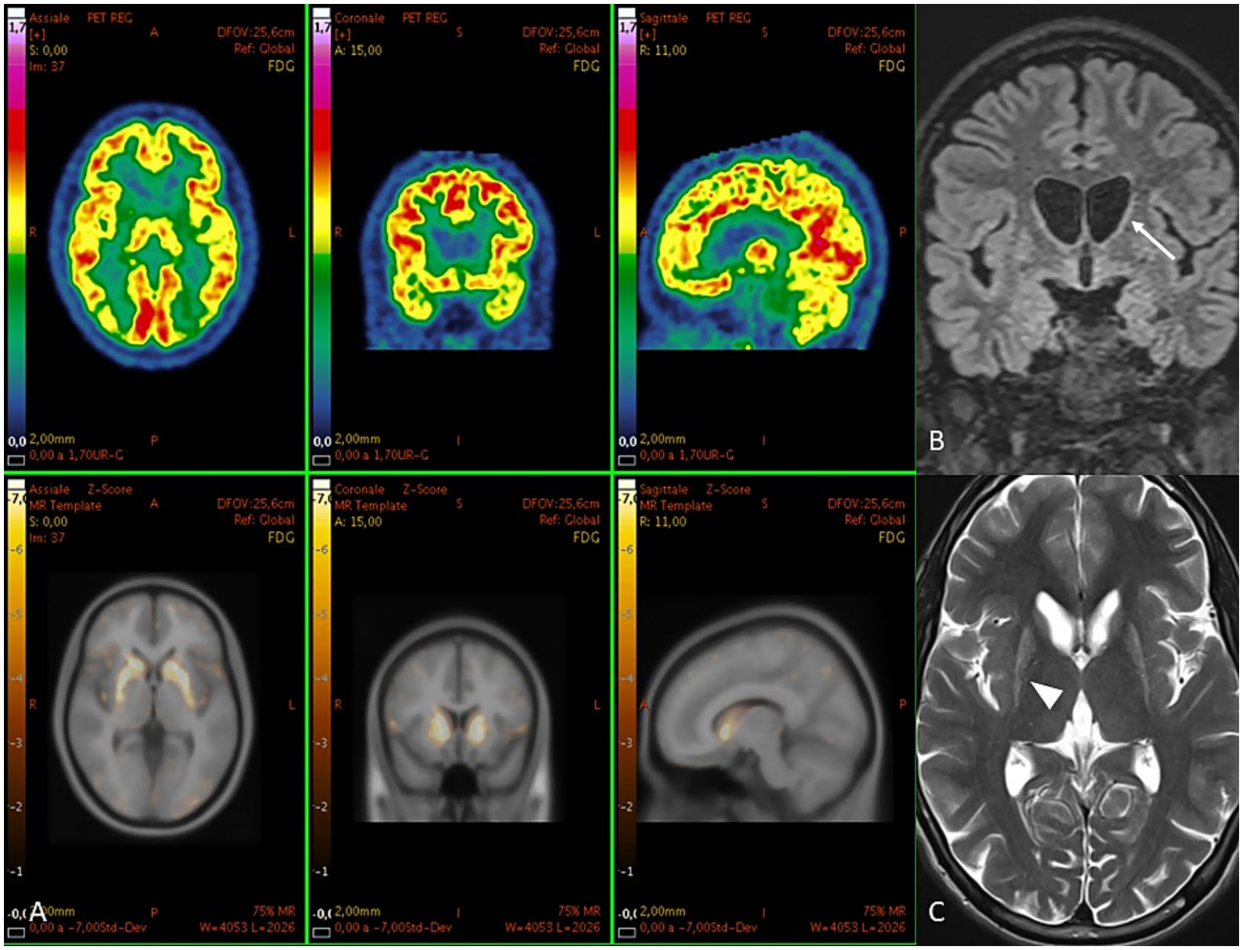
**(A)** PET images in patient two. Above [18F] FDG images and below their *z*-score maps show reduced uptake in caudates and putamina. At MRI, **(B)** coronal FLAIR shows a symmetric flattening of the lateral profile of the frontal horn due to atrophy of the caudate head (arrow). **(C)** axial T2 shows hyperintense and atrophic putamina (arrowhead).

A positron emission tomography (PET) scan with 18F-FDG on qualitative analysis documents normal distribution of the radiopharmaceutical in all cortical areas, the cerebellum, and the thalami, with almost absent uptake in caudates and putamina bilaterally. MRI shows a symmetric flattening of the lateral profile of the frontal horn due to atrophy of the caudate head and T2 hyperintense and atrophic putamina. See Table 1 and Figure 3 for further details.

## 4 Discussion

In this exploratory study, we investigated the hypothesis of a potential dysfunction of the glucose transporter GLUT1 in pediatric Huntington’s disease (PHD), following recent findings from Tramutola et al. (5), who reported markedly reduced GLUT1 expression in the striatum and frontal cortex of PHD patients. These alterations were proposed to underlie clinical parallels between PHD and GLUT1 Deficiency Syndrome (GLUT1DS), including seizures and movement disorders.

Although our metabolic evaluation in two genetically confirmed PHD patients did not reveal a biochemical profile consistent with GLUT1DS one patient displayed a borderline CSF/plasma glucose ratio. While still within or near the lower normal range, such borderline values—especially in the context of rare diseases—warrant cautious interpretation and ideally require confirmation through repeated measurements.

While we found no evidence of systemic glucose transport impairment, PET imaging revealed severe hypometabolism localized to the basal ganglia, supporting previous neuropathological and neuroimaging studies that point to region-specific energy dysregulation in PHD (13, 14).

Both patients’ EEGs demonstrated diffuse background slowing, accompanied by epileptiform activity that increase during sleep, with no correlation observed between these findings and fasting status. These results align with existing literature, reflecting the electroclinical progression of pediatric Huntington’s disease (15). In contrast, GLUT1DS typically presents with generalized EEG slowing that becomes more pronounced during fasting (16). Additionally, in some cases, fasting EEG reveals diffuse spike–wave discharges that markedly improve following food intake (17), indicating a direct impact of metabolic dysfunction on cerebral electrical activity.

Taken together these findings do not support a generalized systemic GLUT1 deficiency in PHD, but rather highlight a central, brain-region specific glucose metabolism impairment.

At present this does not justify the use of metabolic interventions such as the ketogenic diet in PHD subjects.

Dysregulated glucose metabolism within the basal ganglia—particularly the caudate and putamen—appears to play a central role in driving both neurodegeneration and clinical manifestations in pediatric-onset Huntington’s disease (PHD). Functional imaging reveals marked glucose hypometabolism in these nuclei, evident even before overt atrophy and clinical symptoms emerge; the degree of hypometabolism correlates with CAG repeat expansion and disease severity, indicating a pathogenic link between early metabolic failure and structural degeneration (9, 18, 19). Hybrid PET/MRI studies in pediatric-onset HD have shown that regions with more severe striatal volume loss also display disproportionately reduced glucose uptake, aligning metabolic impairment with structural vulnerability and hypokinetic motor phenotypes, in contrast to the choreic features typical of adult-onset cases (20). Mechanistically, deficient glucose uptake—driven by reduced GLUT3 expression in striatal neurons and altered glycolytic enzyme activity, such as phosphofructokinase and pyruvate dehydrogenase—leads to energy deficits that compromise neuronal integrity and synaptic maintenance (5, 21). This metabolic stress is further amplified by mitochondrial dysfunction, particularly complex II deficits and impaired oxidative phosphorylation, which increase oxidative damage and promote neuronal loss in the striatum (7, 8). Structural declines in basal ganglia volume and corticostriatal connectivity, as shown by diffusion-tensor imaging and volumetric MRI, closely align with both motor and cognitive impairments, reinforcing the view that metabolic deficits are upstream drivers of neurodegeneration and clinical phenotype expression in PHD (22). Thus, basal ganglia hypometabolism emerges as both a driver and a biomarker of striatal atrophy and the hypokinetic–dystonic clinical presentations observed in PHD.

Despite the very limited sample size, our study adds to the growing body of literature implicating altered energy metabolism in PHD.

We recommend including CSF glucose analysis as part of the diagnostic workup in severe or early-onset PHD phenotypes, particularly to identify potential metabolic subgroups who may benefit from tailored interventions. However, larger studies with age-matched controls and repeated metabolic assessments are essential to validate these preliminary findings and to allow for statistical comparisons.

## Data Availability

The raw data supporting the conclusions of this article will be made available by the authors, without undue reservation.
